# Are there double knots in proteins? Prediction and *in vitro* verification based on TrmD-Tm1570 fusion from *C. nitroreducens*

**DOI:** 10.3389/fmolb.2023.1223830

**Published:** 2024-06-06

**Authors:** Agata P. Perlinska, Mai Lan Nguyen, Smita P. Pilla, Emilia Staszor, Iwona Lewandowska, Agata Bernat, Elżbieta Purta, Rafal Augustyniak, Janusz M. Bujnicki, Joanna I. Sulkowska

**Affiliations:** ^1^ Centre of New Technologies, University of Warsaw, Warsaw, Poland; ^2^ Polish-Japanese Academy of Information Technology, Warsaw, Poland; ^3^ Faculty of Chemistry, University of Warsaw, Warsaw, Poland; ^4^ Laboratory of Bioinformatics and Protein Engineering, International Institute of Molecular and Cell Biology in Warsaw, Warsaw, Poland

**Keywords:** methyltransferase, composite knot, SPOUT, domain, evolution

## Abstract

We have been aware of the existence of knotted proteins for over 30 years—but it is hard to predict what is the most complicated knot that can be formed in proteins. Here, we show new and the most complex knotted topologies recorded to date—double trefoil knots (3_1_ *#*3_1_). We found five domain arrangements (architectures) that result in a doubly knotted structure in almost a thousand proteins. The double knot topology is found in knotted membrane proteins from the CaCA family, that function as ion transporters, in the group of carbonic anhydrases that catalyze the hydration of carbon dioxide, and in the proteins from the SPOUT superfamily that gathers 3_1_ knotted methyltransferases with the active site-forming knot. For each family, we predict the presence of a double knot using AlphaFold and RoseTTaFold structure prediction. In the case of the TrmD-Tm1570 protein, which is a member of SPOUT superfamily, we show that it folds *in vitro* and is biologically active. Our results show that this protein forms a homodimeric structure and retains the ability to modify tRNA, which is the function of the single-domain TrmD protein. However, how the protein folds and is degraded remains unknown.

## 1 Introduction

The presence of knots in proteins has been known for almost 30 years, but only a few types of knots were identified over the years: 3_1_, 4_1_, 5_2_, and 6_1_ (Eriksson et al., 1988; Taylor, 2000; Wagner et al., 2005; Das et al., 2006; Schmidberger et al., 2008; Bölinger et al., 2010; Thiruselvam et al., 2017; Sulkowska, 2020). The most commonly encountered knot is the trefoil (3_1_), which is found in more than 85% of knotted structures. However, the number of knotted proteins is not high (185) which makes up about 0.3% of all the proteins with structures deposited in the PDB database (Jamroz et al., 2015). This raises at least two fundamental questions. First, why is the number of knotted proteins so low, even though it is expected from polymer physics to be much higher (Lua and Grosberg 2006)? It could be due to the complicated folding process needed to form the knot, including threading, which is energetically costly (Yeates et al., 2007), or maybe entanglement is disadvantageous in the process of protein degradation. However, since knotted domains frequently contain binding sites for substrates (Sulkowska, 2020), their presence is essential for certain proteins, such as methyltransferases (MTs) (Tkaczuk et al., 2007; Christian et al., 2016; Perlinska et al., 2020b). The second question is why proteins can only form simple types of knots? Why not more complex knots with more crossings or multiple separate knots since multi-domain proteins do exist? Both questions are not trivial to answer. Herein, we will try to solve the second one.

**FIGURE 1:**
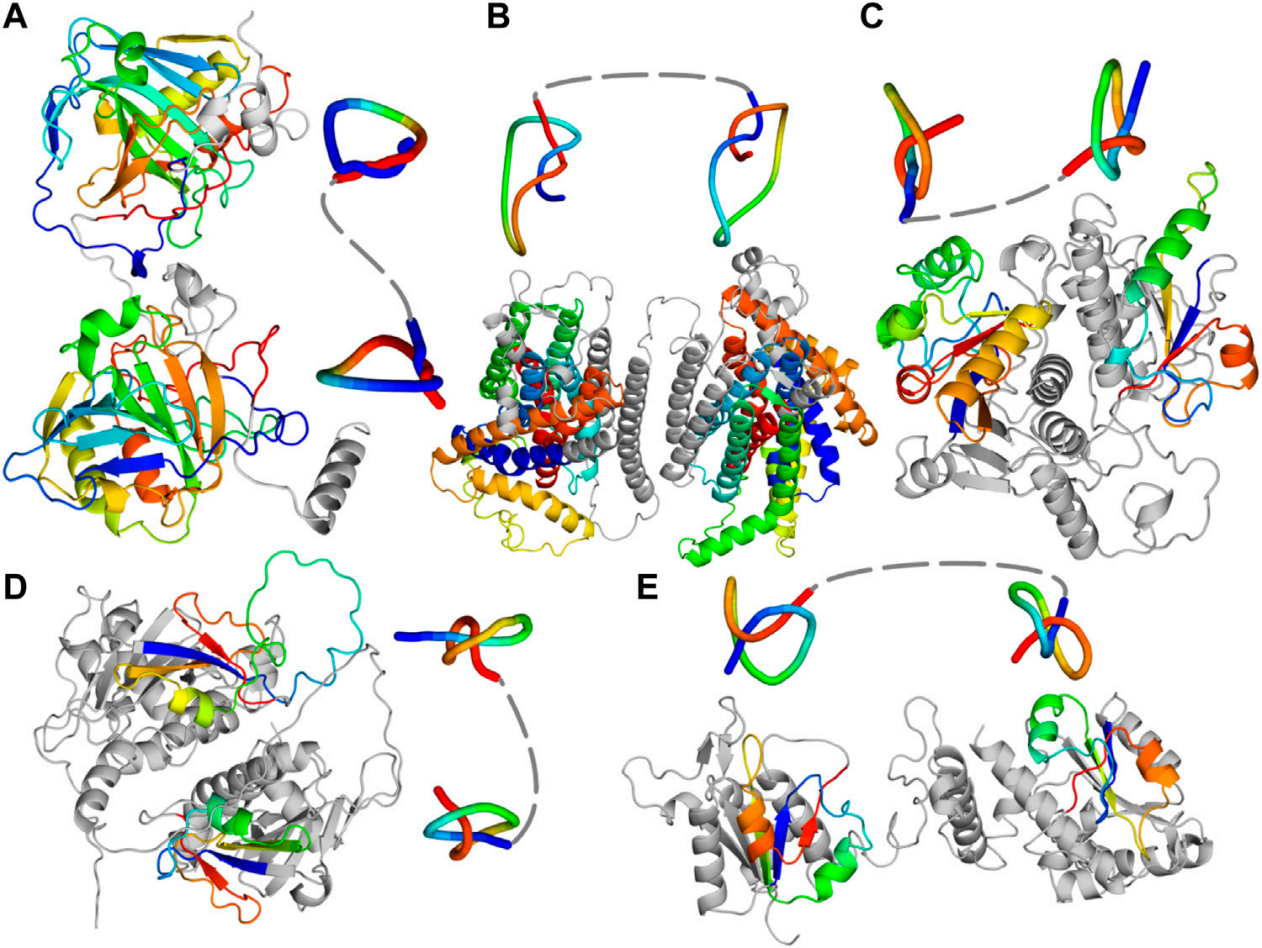
Predicted single chain structures of the fusion proteins. **(A)** Carbonic anhydrase (PF00194-PF00194; UniProtKB ID: A0A0B7AKD5) **(B)** Protein from Ca^2+^: Cation Antiporter (CaCA) family (UniProtKB ID: A0A0L0BYW8). **(C)** Protein with PF00588-PF00588 architecture (UniProtKB ID: Q4DMW6). **(D)** Nep1-Nep1 protein (PF03587-PF03587 architecture; UniProtKB ID: A0A498KD62). **(E)** TrmD-Tm1570 protein (PF01746-PF09936 architecture; UniProtKB ID: E4THH1). All the models were predicted with AlphaFold 2. Knotted regions are shown with rainbow coloring and their reduced structures were obtained with Knot_pull package (Jarmolinska et al., 2020).

In this paper, we expand our knowledge of protein structure complexity and possible folds by presenting newly identified families of proteins with two separate knots (a so-called composite knot; Table 1). For each family, we determined the structures of doubly knotted proteins using AlphaFold 2 and RoseTTaFold (Figure 1), and in the case of one family (TrmD-Tm1570), we performed a more in-depth *in silico* and *in vitro* study based on *Calditerrivibrio nitroreducens*. All of the proteins with composite knots that we found have two 3_1_ knots [based on AlphaKnot database (Niemyska et al., 2022)], which is expected by trefoil’s prevalence in the knotted proteins world (Dabrowski-Tumanski et al., 2019). In our search, we have not identified any other structures involving two sequential knots, other than two trefoils. However, just as the single-knotted proteins, the composite knots are also not commonly found in a proteome, but the scale is different—a well-studied knotted MT called tRNA methyltransferase D (TrmD) is a universal protein for all Bacteria (Ito et al., 2015; Zhong et al., 2019), but the presence of proteins with composite knots is limited to specific organisms, such as *Desulfovibrio vulgaris* or *Oleidesulfovibrio alaskensis*.

**TABLE 1:** Proteins with composite knots.

Superfamily	Fusion architecture (Pfam ID)	No. proteins	Example protein (UniProtKB ID)	Knot type
Carbonic anhydrase	PF00194-PF00194	686	A0A0B7AKD5	3_1_ *#*3_1_
CaCA	PF01699-PF01699-PF01699-PF01699	24	A0A179I9N9	3_1_ *#*3_1_
SPOUT	PF00588-PF00588	125	Q4DMW6	3_1_ *#*3_1_
SPOUT (Nep1-Nep1)	PF03587-PF03587	34	A0A498KD62	3_1_ *#*3_1_
SPOUT (TrmD-Tm1570)	PF01746-PF09936	66	E4THH1	3_1_ *#*3_1_

The majority of the proteins with composite knots represent the SPOUT superfamily, although in most cases the recognition of specific proteins behind that architecture was not possible. Reported Pfam IDs are of entangled domains.

Thus we showed that proteins can adopt more complex structures. So, the rarity of knotted proteins might be connected with their complex topology and folding pathway. The knots in proteins (with determined 3D structure) identified to date are exclusively of twist type (Dabrowski-Tumanski et al., 2019), which means that the rate-limiting threading during protein folding occurs only once (Taylor, 2007; Yeates et al., 2007; Bölinger et al., 2010; Sulkowska, 2020). With structures containing two knots (regardless of their type), the threading must happen at least twice, which is a substantial energetic cost for the cell. Therefore, doubly knotted proteins may provide additional benefits over single-knotted proteins to be advantageous for the cell (Dabrowski-Tumanski et al., 2016b; Sulkowska, 2020). This advantage might be connected to the reason for many other proteins fusing together—these proteins are interacting and forming functional complexes while remaining separate (Chia and Kolatkar, 2004; Pasek et al., 2006; Buljan et al., 2010; Marsh et al., 2013). Thus, when their genes are nearby or are fused, they can be expressed together (both in time and place), which is energetically favorable.

Herein, having discussed the general characteristics of four of the five doubly knot families, we focus on one family of doubly knotted proteins—a TrmD-Tm1570 fusion, which joins two domains with MT activity. Both of the domains function also as single proteins (Tkaczuk et al., 2007; Kim et al., 2009; Perlinska et al., 2020a). To better understand the structure, evolution and biological function of this fusion on doubly knotted TrmD-Tm1570 from *C. nitroreducens* (*Cn*TrmD-Tm1570), we conducted an *in vitro* study of this protein’s activity and an extensive bioinformatics analysis of sequences and structures of its related proteins. Our results show that the *Cn*TrmD-Tm1570 protein is a functional homodimeric protein that methylates tRNA, and thus the *Cn*TrmD domain is fully operational (Christian et al., 2016). However, the function of the second domain (*Cn*Tm1570) is not clear except that it is similar to 2’O ribose-modifying knotted MTs (Kim et al., 2009). Moreover, how the double knotted proteins fold, and whether they can be easily degraded are going to be new open fundamental questions.

## 2 Materials and methods

### 2.1 Search for double knotted proteins

First, we obtained information about domains found in entangled protein structures from the KnotProt database (Dabrowski-Tumanski et al., 2019). Next, we calculated the minimal length of each domain, which was based on the shortest knotted region of the domain. This step was important for finding proteins long enough to be doubly knotted. The implicated domains were classified into three groups based on their contribution in creating a knot, slipknot, or both. We then retrieved all the sequences present in the Pfam database (Mistry et al., 2021), which consisted of at least two entangled functional regions, and created a list of proteins divided by their domain architecture. We selected the proteins with the highest possibility of containing a composite knot. From each domain architecture type, we chose a few sequences (also regarding their length). We considered representative proteins to form a composite knot when their closest homolog with a known 3D structure contained at least one entanglement. Therefore, we identified the most similar sequences within the AlphaFold Protein Structure Database using the Basic Local Alignment Search Tool (BLAST) in ChimeraX 1.3 (Pettersen et al., 2021). The best hit was checked based on its e-value and presence in the entanglements. If the e-value was significant (*e* − *value* < 10^–3^) and the homolog turned out to be knotted, the examined protein was modeled in AlphaFold 2. It is worth noting that all found homologs had e-values between 1e-07 and 0.0 (with most being between 1e-30 and 0.0). Obtained structures were additionally analyzed using the AlphaKnot server.

### 2.2 Dataset preparation and quantification of dimer interfaces

The PDB (Berman et al., 2000) was scanned for entries representing TrmD as well as Tm1570 in different source organisms with a resolution better than 4.0 Å. We identified the pairwise interactions and quantified the interface area (B) using the following equation:

$$B=SASA_{1}+SASA_{2}-SASA_{12}.$$ (1)

Here, the first two terms represent the solvent accessible surface area (SASA) of the molecules in isolated form and the last term represents the SASA of their binary complex. SASA values were calculated using the program NACCESS (Hubbard and Thornton, 1993), which implements the Lee and Richards algorithm (Lee and Richards, 1971).

### 2.3 Sequence analysis

The sequences of fusion proteins were retrieved from the UniProt Knowledge base (UniProtKB) (Consortium, 2021), by searching for both protein families of TrmD (Pfam id: PF01746) and Tm1570 (PF09936) proteins. Then the sequences were clustered using the CD-HIT suite (Huang et al., 2010) (three runs, with sequence identity cutoff ranges from 0.9 to 0.7). Multiple sequence alignment was done using Clustal Omega (Madeira et al., 2022) and the conservation of residues was analyzed using UGENE (Okonechnikov et al., 2012).

### 2.4 Molecular docking

The tRNA from *Haemophilus influenzae* (*Hi*TrmD) (PDB id: 4YVI) was extracted and docked to the TrmD domain of the fusion protein with known constraint (tRNA binding motif) using the HDOCK server (Yan et al., 2017). The C*α* root mean square deviation (RMSD) to the PDB structure 4YVI was used to filter the fusion-tRNA complexes, and the best superposed complex was chosen for further study. Following that, four ligands (S-adenosylmethionine; SAM) were docked to the respective knotted domains of TrmD and Tm1570 in the fusion-tRNA protein-RNA complex using the GLIDE program in the Schrődinger software (Maestro 12.5). The fusion-tRNA structure was prepared using the Protein Preparation Wizard. Prior to docking to Tm1570 we added a water molecule, which based on Tm1570 crystals, is important for a proper pose of SAM. We also rotated the side chain of Asn409 accordingly to Tm1570 crystal structures and minimized the protein, in order for the residue not to block the binding site. Docking to Tm1570 was performed with H-bond constraints defined on the amine group of Ala357 and the backbone oxygens of Ile402 and Asn409.

### 2.5 Structure prediction with AlphaFold and RoseTTaFold cross-validation

Structures were predicted with our locally installed newest AlphaFold 2 version (2.1.0). Proteins with potential composite knots based on the AlphaFold 2 calculations were validated on the RoseTTaFold server (Baek et al., 2021) using default parameters. Since RoseTTaFold does not accept sequences with more than 1,200 residues, the larger proteins were modeled within this range. All the modelled structures were deposited in the Github repository (https://github.com/ilbsm/Double_knots_structures/tree/main).

### 2.6 Experimental procedures

Codon-optimized gene coding for TrmD-Tm1570 was synthesized (GenArt, Thermo Scientific) and inserted into the pET28b expression vector. Transformed *E. coli* BL21 (DE3) RIL cells were grown at 37°C until OD_600_ reached 0.8. After induction with 1 mM IPTG the temperature was decreased to 18°C and bacteria were harvested 18 h later. The cell pellet was resuspended in a lysis buffer containing 6 M urea, 20 mM Hepes, 300 mM NaCl, 10 mM imidazole and 0,01% NaN3. The protein was purified in a HisTrap HP 5 mL column (GE Healthcare). After elution, the protein was dialyzed for at least 5 h against 2 L of buffer (50 mM Hepes, 300 mM NaCl, 20% glycerol, 0.01% NaN3, 2 mM DTT, pH 7.4) in each concentration of urea containing 2 M, 1 M, 0.8 M, 0.6 M, 0 M (5 different buffers). The final purification step involved a preparative Superdex 75 column with the running buffer containing 50 mM Hepes, 300 mM NaCl, 10% glycerol, 1 mM TCEP, pH 7.4. The pure protein was flash-frozen in liquid nitrogen and stored at −80°C. Activity assays were performed with the MTase-Glo kit (Promega) according to the manufacturer’s instructions. The luminescence was performed on a Synergy H1 plate reader (Biotek). Further details are given in SI Materials and Methods.

## 3 Results

Our search for composite knots in proteins is based on two approaches, with direct and indirect use of available protein structures. The indirect approach is focused on the protein sequence and domain annotation. We use the KnotProt database (Dabrowski-Tumanski et al., 2019) and the information it contains about knotted proteins along with the location of the knot in their structures. We associate the presence of a knot with the domain in which it is located. With this data, we searched for multi-domain proteins with more than one entangled domain. For this purpose, we use the Pfam database that contains protein domains grouped into families and superfamilies. This resulted in finding five domain architectures that could possess double knots (Table 1).

In our second approach, we analyze the available protein structures directly. Thanks to the ever-growing number of predicted models with the machine learning methods like AlphaFold (Jumper et al., 2021; Varadi et al., 2022) and our AlphaKnot database that analyses their topology (Niemyska et al., 2022), we were able to find proteins with double knots based on their 3D structure. In order to minimize the probability of finding artifacts, we used only models predicted with high confidence (pLDDT score). We did not encounter any other fusion architectures but found seven additional proteins for the PF00588-PF00588 architecture, that we already found with the first method (Table 1).

Finally, we used locally installed AlphaFold 2 (original model; 2.1.0) to predict the structures of selected proteins (at least two members from each family). Moreover, for additional verification, we used RoseTTaFold to predict structures for the same protein sequences and compare their topologies. The example doubly knotted protein from each of the architectures is presented in Figure 1 and Table 1. The other proteins with predicted doubly knotted structures are shown in the Supplementary Table S1).

All of the architectures we found represent composite 3_1_ *#*3_1_ knots, which is expected since the trefoil (3_1_) knot is the most common knot type found in proteins. As anticipated, the majority of the architectures are formed by domains from the SPOUT superfamily since it is the biggest group of deeply knotted 3_1_ proteins (Sulkowska, 2020). Moreover, we found composite knots also within membrane proteins, namely, the ion transporters.

### 3.1 Carbonic anhydrases

A substantial number of composite knots is found within carbonic anhydrases (Sayre et al., 2011; Dzubiella, 2013). This is a well-known group of knotted proteins with many structures resolved experimentally. Most of these enzymes have a single domain (PF00194) that contains either a deep or a shallow 3_1_ knot (human carbonic anhydrase IX and II, respectively). The fusion of two such domains results in a double 3_1_ knotted protein, with a knot encompassing most of the structure (Figure 1A). We found this architecture (PF00194-PF00194) in 686 protein sequences (131 AlphaFold models with 3_1_ *#*3_1_ can be found in the AlphaKnot database. The difference in the number may be due to the fact that several sequences may be too short to form double knot). Importantly, carbonic anhydrases can form disulfide bridges. These bridges can be formed both within a single chain, thereby creating a lasso that stabilizes a shallow knot (Dabrowski-Tumanski et al. 2016a; Niewieczerzał and Sulkowska 2019; Niemyska et al., 2020). They can also be formed between the monomers forming a dimer (Alterio et al. 2009; De Simone and Supuran 2010).

### 3.2 The Ca^2+^: Cation Antiporter (CaCA) family—transmembrane protein

This family of membrane transporters already contains known knotted proteins, such as Vacuolar cation/proton exchanger (CAX) with a 3_1_ knot (Jarmolinska et al., 2019). This protein has two PF01699 domains that together form a knot. Here, we found proteins with four such domains which indicate that they could possess two knots within their structure. This architecture (PF01699-PF01699-PF01699-PF01699) is present in 24 proteins (Table 1) and none of them has a resolved structure. Figure 1B shows a structure we predicted with AlphaFold (RoseTTaFold also predicted two knots in this protein). Other proteins from this group with the potential to be doubly knotted are listed in the supplement (Supplementary Table S1).

### 3.3 The SPOUT family

Apart from the TrmD-Tm1570 proteins that we describe in more detail below, the SPOUT family contains at least two different architectures also forming a composite 3_1_ *#*3_1_ knot (Table 1). Most probably they are a result of gene duplication—the gene coding the knot-containing domain fused with its duplicate resulting in a double knotted protein. Individual SPOUT families (Pfam ID) shown in Table 1 represent the MT domain, which functions as a single protein in many organisms (Hou et al., 2017). The common structural feature among all of the structures we predicted is the fact that the duplicated domains are interacting *via* the vast interface (Figures 1C, D). Furthermore, the arrangement of the domains is identical to that formed by single-domain proteins in their homodimeric complex (Supplementary Figure S1).

#### 3.3.1 PF00588-PF00588—the largest group of doubly knotted proteins

The PF00588 is a large protein family with over 35,000 sequences of MTs modifying 2’O of ribose in either tRNA or rRNA. The PF00588-PF00588 fusion proteins form the biggest group of the doubly knotted proteins we found. Interestingly, we encountered proteins with this architecture using domain annotations in Pfam (118 hits) and structure searches in AlphaKnot (seven hits). Since these seven proteins do not have both domains annotated in the Pfam database, we used HHpred to obtain them (Supplementary Table S2).

Both domains of this architecture contain a compact 3_1_ knot, which is characteristic of the SPOUT superfamily. All seven models look like a fused dimeric complex of a knotted MT (Figure 1C; Supplementary Figure S1) with the domains arranged in a perpendicular fashion, for example, in TrmH (from the same family) or Tm1570 (from PF09936 family) crystal structures (Kim et al., 2009).

#### 3.3.2 PF03587-PF03587—Nep1-Nep1 fusion

There are 34 proteins with the double knot topology and PF03587-PF03587 domain architecture. The PF03587 family groups Ribosomal RNA small subunit MTs NEP1 (Nep1) that methylate pseudouridine at position 1,189 (Psi1189) in 18S rRNA. Most of these proteins function as homodimeric single-domain proteins. The predicted structure of Nep1-Nep1 fusion we found (Figure 1D) resembles the crystal structure of Nep1 dimeric complex (Supplementary Figure S1)—similar as in the case of PF00588-PF00588 proteins.

#### 3.3.3 PF01746-PF09936—TrmD-Tm1570 fusion

The next group of doubly knotted proteins is formed by the PF01746-PF09936 architecture with both families found within SPOUT knotted MTs. There are a couple of crucial differences between these proteins and the other SPOUT fusions we discussed above: 1) the evolutionary mechanism does not involve gene duplication, 2) in a single chain the two domains interact using a minimal interface, 3) TrmD-Tm1570 is a homodimer, whereas the aforementioned fusion proteins can function as monomers. All of these points are discussed in detail in the sections below.

There are 66 proteins with the PF01746-PF09936 annotation in Pfam and all are present in Bacteria. Within the PF01746 family, there is a well-studied TrmD protein (Ito et al., 2015) that modifies the N1 position of G37 in tRNA. It is a universal bacterial protein that in a vast majority of organisms is a single-domain protein. In the PF01746-PF09936 architecture, TrmD is fused with Tm1570.

The Tm1570 protein belongs to the SAM-dependent RNA MT family (Pfam ID: PF09936). It contains only 299 proteins and one of them, namely, Tm1570 from *Thermotoga maritima*, has been crystallized (PDB ID: 3dcm). Based on the structure similarity search we performed with Dali (Holm and Laakso, 2016) using the crystal structure, the protein is most similar to TrmJ MTs that modify cytidine 32 at the 2’O position in tRNA (Purta et al. 2006) (Supplementary Table S3). Therefore, it is highly probable that proteins with the PF09936 domain bind tRNA and perform methylation of ribose 2’O. Within the whole PF09936 family, two types of domain architecture can be found: a single-domain protein (like Tm1570) or a two-domain protein, which is the TrmD-Tm1570 fusion.

**FIGURE 2:**
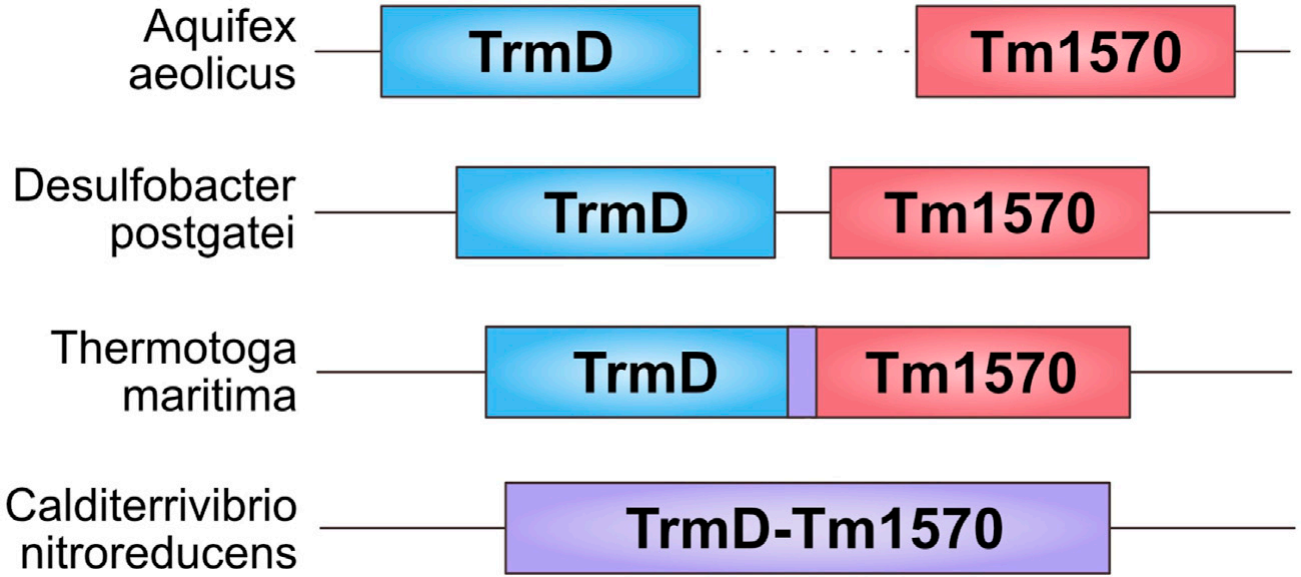
Arrangement of TrmD and Tm1570 genes in different organisms. From the top: *Aquifex aeolicus*—the genes are separated by thousands of nucleotides; *Desulfobacter postgatei*—the genes are 11 nucleotides apart; *Thermotoga maritima*—the genes are overlapping by 7 nucleotides; *Calditerrivibrio nitroreducens*—the genes are fused.

By analyzing the location of the genes coding the domains of both families (Pfam ID: PF01746 and PF09936) in Bacteria, we found four different ways in which the genes are co-located in the genomes: 1) two single genes in different parts of the genome; 2) two single genes adjacent on the genome; 3) two single but overlapping genes; 4) one fused gene. Figure 2 shows the arrangement in some example organisms. This might represent the evolutionary pathway that led to the creation of the fusion, which started as two separate genes. Even though we did not find evidence supporting the interaction between single-domain TrmD and Tm1570, we hypothesize that the proteins can form a complex and it was more advantageous for the cell to express them, and in the end to fuse them, together.

**FIGURE 3:**
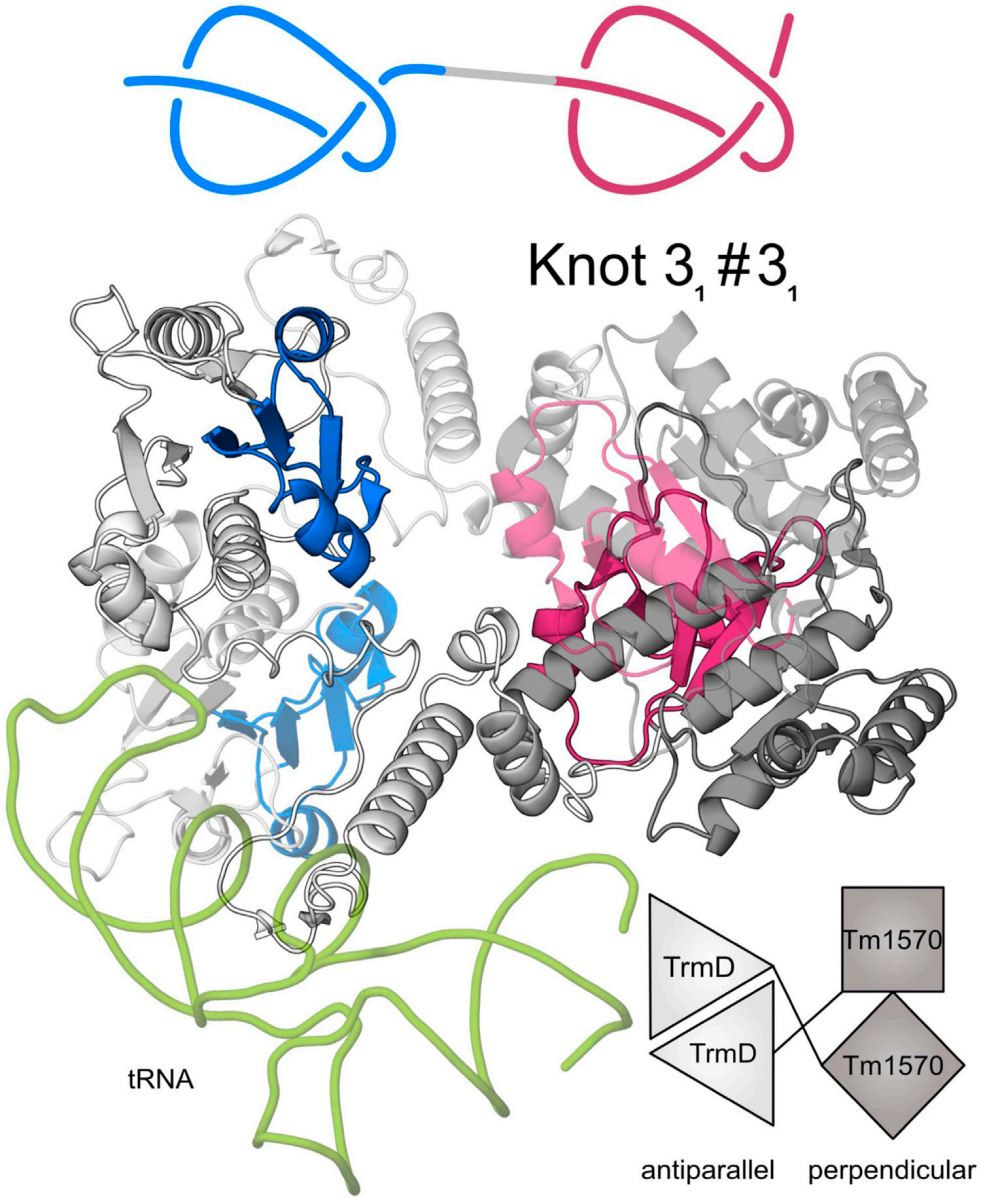
Predicted structure of CnTrmD-Tm1570 fusion protein based on AlphaFold and docking. This homodimeric (second chain is transparent) complex binds tRNA (green) with its TrmD domains. A single chain of this protein contains two 3_1_ knots (marked in blue and red) (Niemyska et al. 2022). TrmD domain (light grey) interacts with its counterpart from the second chain in an antiparallel fashion, whereas Tm1570 (dark grey) in a perpendicular fashion. Details about the modeled complex are in the Methods section.

Next, to verify the presence of the two knots in these proteins, we predicted their structures from nine different organisms (see Supplementary Figure S2) using AlphaFold 2. None of the nine formed a compact single chain structure as is the case in the other families of double knotted proteins from SPOUT superfamily we analyzed (e.g., Nep1-Nep1; Figure 1B). Instead, the nine form an open conformation with the domains scarcely interacting with each other (Figure 1E). This behavior is expected since the two proteins in their single-domain forms are dimerizing in different ways: TrmD in an antiparallel fashion and Tm1570 in a perpendicular fashion (Figure 3). This suggests that TrmD-Tm1570 functions as a dimer and the domains interact with their counterparts from the other chain.

### 3.4 TrmD-Tm1570 from *Calditerrivibrio nitroreducens*

#### 3.4.1 Homodimeric structure

From the set of double knotted proteins we found, we investigated further one from the TrmD-Tm1570 family from *Calditerrivibrio nitroreducens* (*Cn*TrmD-Tm1570). We used this protein to characterize the structural basis for substrate recognition and the overall structural landscape of double knotted proteins.

**FIGURE 4:**
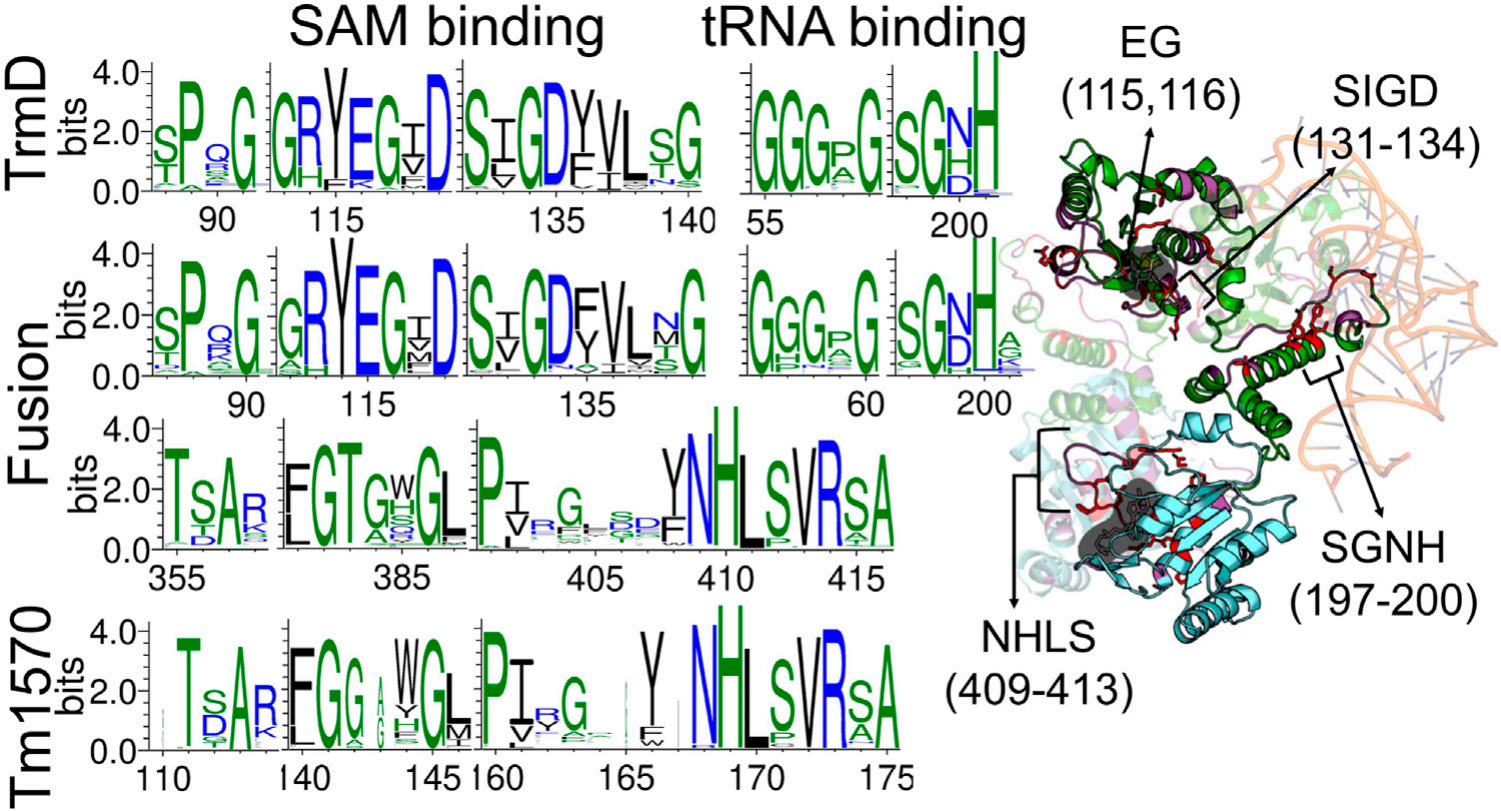
Comparison of sequence motifs in TrmD and Tm1570 with the fusion protein. WebLogo3 was used to construct the conserved residue logos at the SAM and tRNA binding sites. Cartoon representation shows the TrmD-Tm1570 protein (TrmD domain in green, Tm1570 in blue) with selected motifs marked on the structure.

Our theoretical and experimental analysis shows that the *Cn*TrmD-Tm1570 protein functions as a homodimer, unlike other double knotted proteins we study here. Therefore, we used AlphaFold Multimer to model the structure and obtained a compact homodimeric complex (Figure 3) with the main interchain interactions present between the corresponding domains. Both of the domains interact in a standard fashion (antiparallel for TrmD and perpendicular for Tm1570) with their counterparts from the other chain. To further verify the model we analyzed the dimeric interface of the fusion and compared it with the ones that are created in single-domain TrmDs and Tm1570s. We analyzed all TrmD and Tm1570 structures available in the PDB along with their homologs (Supplementary Table S4) and found key residues that are crucial for the dimeric interface in the single-domain proteins (the sequence similarity between different TrmD proteins is low, although, the structures can be superimposed very well). These amino acids are conserved in both the TrmD and Tm1570 domains of the fusion (Figure 4; Supplementary Tables S5, S6).

Details concerning the mechanism of function, including conformations of the residues in the active site as well as the steps of chemical catalysis, are well understood for TrmD (single-domain proteins) but not for Tm1570. Herein, based on our analysis of structure and sequence, we found that deep trefoil knots provide the binding sites for SAM in both the TrmD and Tm1570 domains of the fusion. Moreover, they are both structurally and sequentially similar to their counterparts from single-domain proteins (Figure 4). Thus the ligand binding modes should be similar in both domains. More precisely, the binding site-forming knot in *Haemophilus influenzae* TrmD (*Hi*TrmD) consists of three loops: the cover (Ser88-Gly91), the wall (Gly113-Ile118), and the bottom loop (Ser132-Gly140) (Jaroensuk et al., 2019). The residues of the corresponding three loops in the fusion protein are conserved (the cover loop: Asp87-Gly90, the wall: Gly112- Ile117, and the bottom: Ser131-Gly139). The residues Pro88, Gly90, Arg113, Glu115, Gly116, Ser131, Gly133 and Asp134 are strictly conserved and are involved in the TrmD dimeric interactions (Supplementary Table S6). We used this information to obtain the complex of TrmD-Tm1570 with SAM *via* molecular docking. The resulting structure has four SAM molecules (one ligand in one of the four available active sites), each adopting a conformation that is proper and characteristic for the SPOUT superfamily (Perlinska et al., 2020b) (Supplementary Figure S3).

Finally, based on the known binding mode of tRNA to *Hi*TrmD, we established how the nucleic acid may interact with the *Cn*TrmD-Tm1570 dimer. We found that residues involved in *Hi*TrmD-tRNA interactions are also involved in the fusion (Figure 4). The SGHH motif (residues 198–201 in *Hi*TrmD) that interacts with G37 in the substrate tRNAs for the methylation process (Ito et al., 2015) is present in SGNH form in the *C. nitroreducens* fusion. All of this information strongly suggests that the predicted model correctly demonstrates how the active complex of TrmD-Tm1570 is constructed.

#### 3.4.2 TrmD-Tm1570 is an active tRNA methyltransferase

**FIGURE 5:**
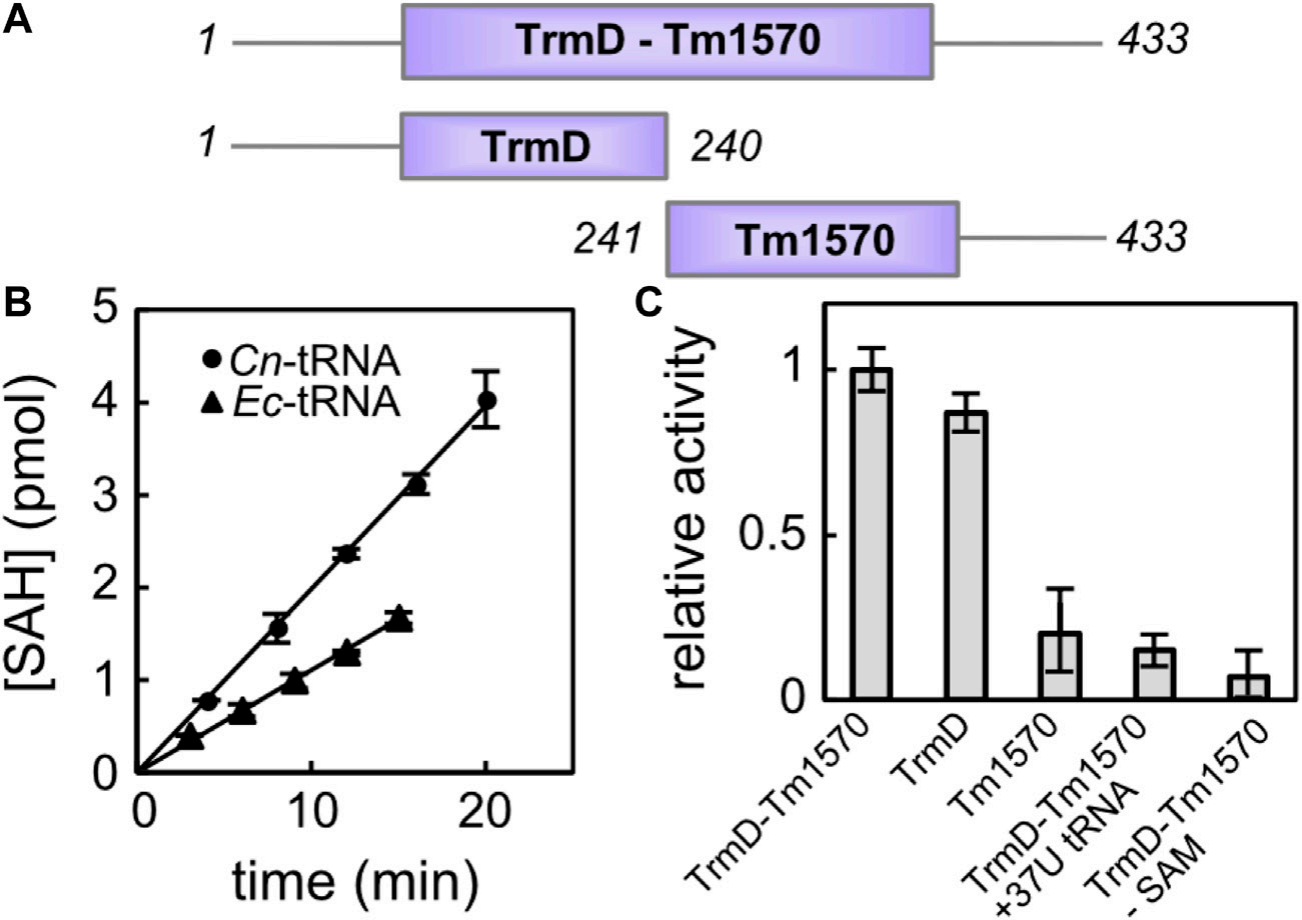
Activity of CnTrmD-Tm1570. **(A)** Protein constructs used for the activity assessment. **(B)** Time-course of the reaction catalyzed by fusion TrmD-Tm1570 (50 nM) in the presence of SAM (30 *μ*M) towards the 8 *μ*M tRNA Leu (CAG) substrate (*E. coli* tRNA black triangles, *C. nitroreducens* tRNA black circles). **(C)** Relative activities of different TrmD-Tm1570 constructs. Data obtained for the mutated tRNA substrate and negative control are also included. Reaction conditions are the same as in **(B)**.

In order to experimentally assess the activity of the fusion TrmD-Tm1570 from *C. nitroreducens*, we expressed the His-tagged protein in *E. coli* cells. However, the full-length protein, as well as its individual truncated domains encompassing residues 1–240 (TrmD) and 241–433 (Tm1570) (see Figure 5A), was found in the insoluble fractions even when growing bacteria at low temperatures. Nevertheless, we managed to perform purification under denaturing conditions and obtained large quantities of structured and functional proteins after the final refolding step. Size-exclusion chromatography suggests that all fragments form stable dimers given the retention volumes on the preparative Superdex 75 column (GE Healthcare; Supplementary Figure S4): 56 mL for the fusion TrmD-Tm1570 (49.6 kDa monomer), 63 mL for the TrmD (27.5 kDa monomer), and 65 mL for the Tm1570 domain (22.1 kDa monomer). It is noteworthy that the single-domain *Ec*TrmD (28.4 kDa monomer), which was previously characterized as a dimer (Elkins et al., 2003) and was now expressed for the sake of reference, eluted from the same column at 63 mL. Moreover, we were able to obtain small quantities of the soluble Tm1570 protein without unfolding procedures and this protein migrated through the gel filtration column as Tm1570 subjected to *in vitro* renaturation.

We tested the MT activity of the recombinant TrmD-Tm1570 using the commercially available MTase-Glo kit (Promega), which allows one to monitor the S-Adenosyl-L-homocysteine (SAH) concentration build-up during the enzymatic reaction. First, we used tRNA Leu (CAG) from *E. coli* (see Supplementary Figure S5 for exact sequence) as the acceptor of the methyl moiety. It turned out that *Cn*TrmD-Tm1570 fusion protein was able to efficiently modify this cloverleaf structure in the presence of SAM. However, as seen in Figure 5B, the same reaction was almost two times faster when the native *C. nitroreducens* substrate, homologous to *E. coli* tRNA Leu (CAG), was used (see Supplementary Figure S5). Although the key nucleotides, including the G37G38 motif, are conserved in both tRNA sequences, not surprisingly TrmD-Tm1570 shows preference towards its native substrate, originating from the same organism as the protein. To verify the importance of the aforementioned G37G38 motif for the reaction catalyzed by TrmD-Tm1570 we also prepared a mutant of *C. nitroreducens* tRNA Leu (CAG), where the guanosine at position 37 was replaced by thymine at the level of the DNA template. As expected for this substrate, the double knotted enzyme showed no activity, comparable to the negative control lacking SAM in the reaction mixture. The activity towards the mutated substrate is higher (at least twice) than control (Figure 5C).

Since we wanted to examine the contribution of both domains to the overall activity of the fusion TrmD-Tm1570 protein, we followed the reaction catalyzed by truncated versions of the full-length protein. With respect to the native *C. nitroreducens* tRNA Leu (CAG) substrate, the TrmD domain retained 86% ± 4% activity of the fusion protein, while Tm1570 retained only 18% ± 9% (see Figure 5C). These data clearly demonstrate that the enzymatic activity of the fusion TrmD-Tm1570 protein towards tRNA Leu (CAG) substrate is governed by the TrmD domain. However, the Tm1570 domain may contribute to substrate binding and increase the stability of the complex. On the other hand, small activity of the isolated Tm1570, comparable to the negative control which had SAM omitted from the reaction mixture, may suggest that this protein, whose physiological function remains unknown, is a tRNA methyltransferase but with different substrate specificity. It is also possible that Tm1570 regains its full activity in the presence of a specific ligand or under yet-to-be-discovered conditions.

## 4 Conclusion

Answering the question from the title: yes, proteins with double knots do exist. Here, for the first time we found and analyzed in-depth such proteins. All of them have two 3_1_ knots and come from three different protein superfamilies (five distinct architectures). They are either transmembrane ion transporters (from the *Ca* ^2+^: Cation Antiporter family), carbonic anhydrases, or methyltransferases (from the SPOUT family). Within the SPOUT group, there are three architectures in proteins with either two duplicated domains (PF00588-PF00588 and PF03587-PF03587) or two separate domains (PF01746-PF09936—TrmD-Tm1570). We found that these two groups differ in terms of structure organization—the duplicated and fused domains form dimer-like single chain structures, whereas the TrmD-Tm1570 proteins need two chains to resemble a functional SPOUT MT (which are mostly dimers).

Using both theoretical and experimental approaches we studied in detail TrmD-Tm1570—a fusion between TrmD and Tm1570 proteins found in 66 organisms. Based on *C. nitroreducens* we established that the protein is a homodimer capable of binding four ligands (S-adenosylmethionine)—one in each knotted binding site, and a single tRNA molecule (based on the similarities with *Hi*TrmD). Moreover, the *Cn*TrmD-Tm1570 is folding and functioning *in vitro*—it methylates tRNA using its TrmD domain. Unfortunately, we were not able to determine the function of the Tm1570 domain—which is unknown for single-domain proteins as well. However, based on structural similarities, we believe that it also acts as a tRNA MT, probably by modifying 2’O in ribose. During the preparation of this manuscript, we were able to solve experimentally the structure of *Cn*TrmD-Tm1570 protein and deposit it in the PDB (PDB id: 8b1n) (da Silva et al., 2023). Our model and the crystal structure are similar, in particular, both of them show “open” domain organization that enables homodimer formation.

Herein, we have identified proteins with knotted 3_1_ *#*3_1_ structure, composed of two sequential trefoils. A natural question is whether more complicated composite knots also exist in nature. In our search we have not identified any other more complicated sequential pairs (involving other knots than two trefoils). However, another hypothetically possible (albeit quite unlikely) structure involves one trefoil knot formed within another trefoil. Identification of such a structure would require much more sophisticated search; if such a structure exists, it would very likely have even more complex functional properties. Another method would need to be employed to find such a structure, because the methods used in this research are specific to the case of sequential trefoils.

Note that our research was conducted based on the sequences of all known knotted proteins (domains) (Dabrowski-Tumanski et al., 2019), including predicted knotted proteins (Perlinska et al., 2023) based on the results of AlphaFold 2 till the end of June 2022. During the preparation of this manuscript, a paper by Brems et al. (2022) was published showing the composite knots in AlphaFold structures of SPOUT methyltransferases and carbonic anhydrases. Moreover, given that new primary knots are being identified in structures predicted by AlphaFold Niemyska et al. (2022), we predict that different types of composite knots exist in nature, and their identification is an important task for future research.

Finally, let us comment on folding of proteins with 3_1_ *#*3_1_ knots. Based on known folding pathways of proteins from SPOUT family one could imagine knotting the N-terminal chain directly on the ribosome (Chwastyk and Cieplak, 2015; Dabrowski-Tumanski et al., 2018; Baiesi et al., 2019), while the C-terminal knot could follow a well known slipknot pathway (Wallin et al., 2007; Sulkowska et al., 2009) or other mechanisms suggested for proteins with a deep knot based on numerical simulations (Potestio et al., 2010; Li et al., 2012). Another possibility is to use flipping mechanism observed in numerical simulations for proteins with, e.g., 6_1_ knot (Bölinger et al., 2010). This mechanism was later developed to a topological descriptor of knot folding by Flapan et al. (2019). One could extend this scenario to explain folding of newly identified proteins with no-twist types of knots, such as 6_3_ recently predicted based on AlphaFold approach (Perlinska et al., 2022). However, it is not clear how to use it directly to 3_1_ *#*3_1_ knots; moreover, in the case of all doubly knotted proteins listed in this work, we do not see loops responsible for flipping. Reconstructing a folding pathway of composite knots is also an important task for future research.

## Data Availability

The original contributions presented in the study are included in the article/Supplementary Material, further inquiries can be directed to the corresponding author.
