# Supplementary material for: Are there double knots in proteins? Prediction and in vitro verification based on TrmD-Tm1570 fusion from C. nitroreducens
Source: Front Mol Biosci. 2024 Jun 6;10:1223830. doi: 10.3389/fmolb.2023.1223830 (PMC11187310; doi:10.3389/fmolb.2023.1223830)
Supplement: Supplementary file 1 [file DataSheet1.pdf]

frontiers

Supplementary Material

Are there double knots in proteins? Prediction and in vitro verification based on TrmD-Tm1570 fusion from *C. nitroreducens*

Agata P. Perlinska, Mai Lan Nguyen, Smita P. Pilla, Emilia Staszor, Iwona Lewandowska, Agata Bernat, Elżbieta Purta, Rafał Augustyniak, Janusz M. Bujnicki and Joanna I. Sulkowska*
*Correspondence: Joanna I. Sulkowska (jsulkowska@cent.uw.edu.pl)

MATERIALS AND METHODS

Expression and purification of proteins

The DNA sequence coding for the His-tagged TrmD-Tm1570 fusion protein was codon optimized for expression in *E. coli* cells, synthesized by GenScript and inserted into the expression pET28b vector. Truncated versions of the TrmD-Tm1570 were generated using Q5 Site-Directed mutagenesis kit (New England Biolabs). Protein expression was achieved by growing transformed *E. coli* BL21(DE3) RIL cells in LB medium containing 50 mg/L of kanamycin. Proteins were expressed at 18 °C overnight after induction with 1 mM isopropyl-β-D-thiogalactopyranoside (IPTG) when the OD_{600} reached 0.8. For all proteins, the cells were harvested and resuspended in a lysis buffer containing 50 mM Hepes, 300 mM NaCl, 6 M urea, 10 mM imidazole, 0.01% sodium azide, 1 mM DTT, pH 7.4. Cells were disrupted by sonication and the lysate was cleared by centrifugation at 24 000 g for 1 h. The supernatant was loaded onto the 5 mL HisTrap affinity column (GE Healthcare). The protein was washed with at least 5 column volumes of lysis buffer and 5 column volumes of washing buffer containing higher (20 mM) concentration of imidazole. Elution was achieved by applying 5-10 column volumes of the elution buffer (20 mM Hepes, 300 mM NaCl, 6 M urea, 250 mM imidazole, 0.01% sodium azide, 1 mM DTT pH 7.4). Fraction containing TrmD-Tm1570 or its individual domains was extensively dialyzed against 2L of buffer: 50mM Hepes, 300 mM NaCl, 20% glycerol, 0.01% NaN 3, 2mM DTT, pH 7.4, which included 2 M urea. Approximately 4 hours later a dialysis bag was transferred to a fresh buffer containing 1 M urea for overnight dialysis. The following steps involved 0.8 M, 0.6 M (4 hours each) and finally 0 M urea (overnight). All dialysis steps were done at room temperature. Next, the protein was concentrated using centrifugal filters with 30 kDa membrane cut-off (Vivaspin 20, Sartorius) and purified on HiLoad Superdex 75 1660 pg column (GE Healthcare) with the running buffer containing 50 mM Hepes, 300 mM NaCl, 10% glycerol, 1 mM TCEP, pH 7.4. Fractions containing pure protein were identified on SDS-PAGE, flash-frozen on liquid nitrogen and stored at –80 °C. Plasmid encoding *E. coli* TrmD protein was obtained by amplifying an appropriate fragment from the genomic DNA isolated from DH5α strain and inserting into pET28b vector. The protein was expressed and purified according to the same protocol as TrmD-Tm1570 (see above), however, urea was not included in any of the buffers and only one dialysis step was done to remove imidazole prior to the gel filtration.

Preparation of the tRNA substrates by *in vitro* transcription

The double-stranded DNA template coding for *E. coli* tRNA Leu (CAG) provided with a functional promoter was obtained by PCR using bracketing oligonucleotides. The exact sequences of the oligonucleotides as well as the final tRNA products are listed in Figure S10. The DNA template was PCR-amplified with Q5 DNA Polymerase (NEB). The concentrations of F1 and R4 oligonucleotides in the reaction mix was 100 μM whereas lower amounts (1 μM) were used for R2 and F3. After purification

with the PCR Cleanup Kit (New England Biolabs), the DNA product was used as a template for *in vitro* transcription by T7 RNA Polymerase (Thermo Scientific). At the end of the reaction, the tRNA transcript was purified with Monarch RNA Cleanup Kit (New England Biolabs) and the concentration was measured with DeNovix microvolume UV-VIS spectrophotometer. *C. nitroreducens* tRNA Leu (CAG) sequence was retrieved from RefSeq database (Pruitt et al., 2005) and annotated using tRNAscan-SE (Chan et al., 2021). Appropriate DNA oligonucleotides were designed (see Figure S10) and the same protocol as for *E. coli* tRNA was repeated. In order to produce *C. nitroreducens* tRNA Leu (CAG) mutant that would not be modified by TrmD enzyme, G to T mutation into the DNA matrix was introduced at the position 36 of the template DNA. This resulted in G to U mutation in a final tRNA product as shown in Figure S10.

Methyltransferase activity assays

In order to assess the activity of TrmD-Tm1570 and its individual domains we used a commercially available MTase-Glo Assay Kit (Promega). Luminescence measurements were done using Synergy H1 (Biotek) plate reader in black half-area 96-well plates (Greiner, article number 784904). The methyltransferase reaction was carried out at 37 °C but luminescence measurements were done at ambient temperature. The reaction buffer contained 0.1 M Tris, 50 mM KCl, 1 mM EDTA, 4 mM DTT, 5 mM $MgCl_2$, pH 7.25 and was first used to prepare a standard curve of SAH as illustrated on Figure S11A. Standard curve as well as all measurements of the enzyme activities were performed according to the assay kit manual. First, we determined the linear range of the initial velocities by plotting this parameter against enzyme concentrations (see Figure S11B, C). Since Tm1570 demonstrated almost no activity at these conditions, it was omitted from further experiment optimization procedures. For both full length TrmD-Tm1570 and for the isolated TrmD we decided to use 50 nM as enzyme concentration for further reactions. In order to find optimal substrates concentrations for the experiment where one could compare the activities of different TrmD-Tm1570 fragments, we varied tRNA concentration while keeping enzyme and SAM concentrations constant, 50 nM and 30 μM, respectively. As a result, even though very detailed analysis of the kinetic parameters is beyond the scope of this article, we obtained Michaelis-Menten plots for TrmD-Tm1570 fusion protein and for TrmD domain (Figure S12). Overall, both curves are very similar to each other, and the most pronounced differences are visible for low to medium range tRNA concentrations. Therefore for comparing activities of different protein fragments towards a range of substrates we decided to use 8 μM tRNA, 30 μM SAM and 50 nM enzyme.

REFERENCES

Chan, P. P., Lin, B. Y., Mak, A. J., and Lowe, T. M. (2021). tRNAscan-SE 2.0: improved detection and functional classification of transfer RNA genes. *Nucleic Acids Research* 49, 9077–9096

Pruitt, K. D., Tatusova, T., and Maglott, D. R. (2005). NCBI Reference Sequence (RefSeq): a curated non-redundant sequence database of genomes, transcripts and proteins. *Nucleic acids research* 33, D501–D504

Table S1. Topology of proteins with composite knot architectures. The structures are predicted by AlphaFold (either already deposited in the AlphaFold database or modeled with our locally installed version) or RoseTTaFold modeling. We show the quality of the models for both methods using their scores (pLDDT for AlphaFold and confidence score for RoseTTaFold).

Fusion architecture	UniProtKB ID	AlphaFold database (pLDDT)	AlphaFold modeling (pLDDT)	RoseTTaFold modeling (Confidence)
PF01746-PF09936	A0A0D2JLQ7	-	$3_1\#3_1$ (95.39)	$3_1\#3_1$ (0.84)
PF01746-PF09936	A0A4V3HG45	-	$3_1\#3_1$ (95.05)	$3_1\#3_1$ (0.86)
PF01746-PF09936	Q72DU3	$3_1\#3_1$ (94.57)	-	$3_1\#3_1$ (0.84)
PF01746-PF09936	Q313J9	$3_1\#3_1$ (94.39)	-	$3_1\#3_1$ (0.85)
PF01746-PF09936	A0A075WRC8	-	$3_1\#3_1$ (94.33)	$3_1\#3_1$ (0.84)
PF01746-PF09936	A0A6P1ZP81	-	$3_1\#3_1$ (93.46)	$3_1\#3_1$ (0.84)
PF01746-PF09936	A0A3R5V0D4	-	$3_1\#3_1$ (93.12)	$3_1\#3_1$ (0.85)
PF01746-PF09936	E5YA54	-	$3_1\#3_1$ (90.73)	$3_1\#3_1$ (0.79)
PF01746-PF09936	E4THH1	$3_1\#3_1$ (91.60)	-	$3_1\#3_1$ (0.85)
PF01699-PF01699-PF01699-PF01699	A0A179I9N9	-	$3_1\#3_1$ (80.47)	$3_1\#3_1$ (0.78)
PF01699-PF01699-PF01699-PF01699	A0A1R3IMN4	-	$3_1\#3_1$ (74.97)	$3_1\#5_1$ (0.64)
PF00588-PF00588	A4I142	$3_1\#3_1$ (84.95)	-	$3_1\#3_1$ (0.61)
PF00588-PF00588	Q4DMW6	$3_1\#3_1$ (83.82)	-	$3_1\#3_1$ (0.6)
PF00588-PF00588	Q4D5S2	$3_1\#3_1$ (83.34)	-	$3_1\#3_1$ (0.61)
PF00588-PF00588	Q4CYG6	$3_1\#3_1$ (80.77)	-	$3_1\#3_1$ (0.6)
PF00588-PF00588	Q381U1	$3_1\#3_1$ (80.62)	-	$3_1\#3_1$ (0.6)
PF00588-PF00588	Q4D7N4	$3_1\#3_1$ (80.26)	-	$3_1\#3_1$ (0.6)
PF00588-PF00588	A4I7Y6	$3_1\#3_1$ (70.74)	-	$3_1\#3_1$ (0.57)
PF03587-PF03587	A0A6A6LPQ1	-	$3_1\#3_1$ (72.88)	$3_1\#3_1$ (0.69)
PF03587-PF03587	A0A498KD62	-	$3_1\#3_1$ (68.96)	$3_1\#5_1$ (0.73)
PF00194-PF00194	A0A0B7AKD5	$3_1\#3_1$ (88.96)	-	-
PF00194-PF00194	A0A0L0CGV1	$3_1\#3_1$ (91.85)	-	-
PF00194-PF00194	A0A3Q3AXU9	$3_1\#3_1$ (88.70)	-	-
PF00194-PF00194	A0A210PIM0	$3_1\#3_1$ (88.15)	-	-
PF00194-PF00194	A0A3Q1J491	$3_1\#3_1$ (87.90)	-	-
PF00194-PF00194	A0A7S4L0Y0	$3_1\#3_1$ (86.24)	-	-
PF00194-PF00194	A0A834Z9Q4	$3_1\#3_1$ (88.64)	-	-
PF00194-PF00194	A0A7R8YLY2	$3_1\#3_1$ (87.22)	-	-
PF00194-PF00194	A0A7S3VMD7	$3_1\#3_1$ (86.16)	-	-
PF00194-PF00194	Q84NF2	$3_1\#3_1$ (78.99)	-	-

Table S2. Domain annotations for double knotted proteins from AlphaFold database. Results based on HHpred search of a sequence of a single protein against Pfam v. 35.

Protein ID	N-terminal domain				C-terminal domain			
	Pfam ID	Probability	E-value	Location (aa)	Pfam ID	Probability	E-value	Location (aa)
Q4DMW6	PF00588	99	1.90E-11	35-185	PF00588	98	5.60E-06	255-407
Q4D5S2	PF00588	99	1.40E-11	35-185	PF00588	98	7.40E-06	255-407
A4I142	PF00588	99	1.80E-11	36-179	PF00588	98	8.60E-06	249-401
Q4CYG6	PF00588	99	1.00E-09	53-212	PF00588	96	1.20E-02	317-469
Q4D7N4	PF00588	99	9.20E-10	53-212	PF00588	97	1.00E-02	317-469
A4I7Y6	PF00588	98	8.00E-07	171-276	PF00588	93	2.80E-01	430-582
Q381U1	PF00588	99	9.10E-10	53-212	PF00588	96	1.40E-02	321-473

Table S3. PDB entries that are the most structurally similar to Tm1570 crystal (PDB ID: 3dcm). Based on DALI search against all PDB.

PDB ID	Pfam ID	Z-score	C-alpha RMSD (Å)	No. aligned res (% identity)	No. res in target
4cnf-A	PF00588	16.1	2.2	138 (22%)	155
3kty-A	PF00588	16.1	2.6	148 (17%)	167
5gm8-A	PF00588	15.8	2.3	144 (19%)	171
4cng-B	PF00588	15.6	2.2	135 (22%)	149
3ilk-B	PF00588	15.4	2.4	146 (22%)	233

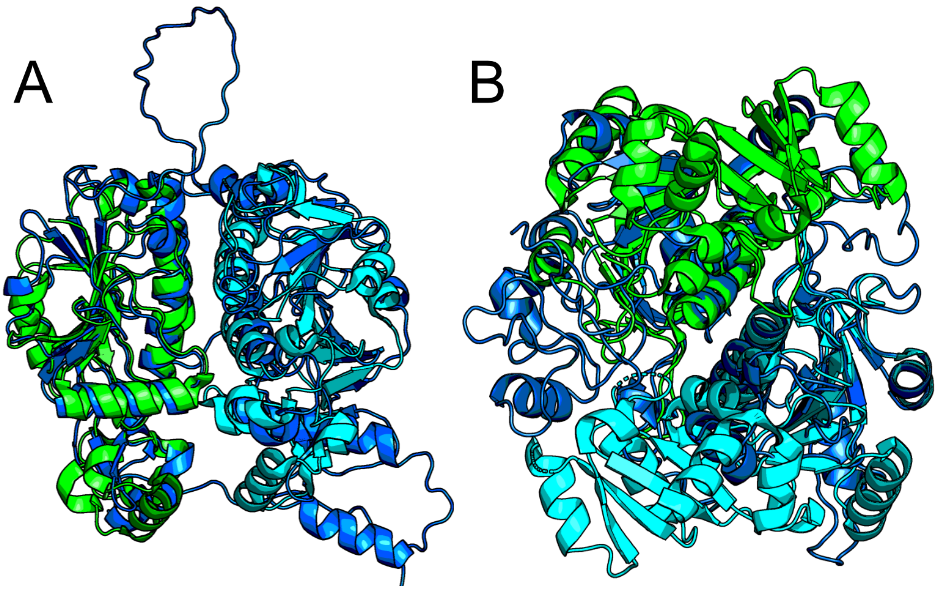

Figure S1: Superposition of the example fusion proteins from PF03587-PF03587 and PF00588-PF00588 architectures with their single-domain counterparts. A. Nep1-Nep1 protein (blue; PF03587-PF03587 architecture; UniProtKB ID: A0A498KD62) with homodimeric structure of Nep1 (green and cyan; PDB ID: 3o7b). B. Protein with PF00588-PF00588 architecture (UniProtKB ID: Q4DMW6) superposed with homodimeric structure from PF00588 family (PDB ID: 3kty). Both of the fusion proteins are predicted to have their domains arranged in the same fashion as single-domain protein form their homodimeric complex.

Table S4. The dataset of TrmD dimers

PDB ID	Source Organism
1P9P	*Escherichia coli*
3IEF	*Bartonella henselae*
3KNU	*Anaplasma phagocytophilum*
3KY7	*Staphylococcus aureus*
4H3Y	*Paraburkholderia phymatum*
4YVI	*Haemophilus influenzae*
5WYQ	*Pseudomonas aeruginosa*
5ZHL	*Mycobacterium tuberculosis*
6QOS	*Mycobacteroides abscessus*
6W14	*Mycolicibacterium smegmatis*
7KFF	*Corynebacterium diphtheriae*
7MYQ	*Acinetobacter baumannii*

Table S5. The residues involved in the dimer contact of TrmD dimers as well as Tm1570 dimer.

TrmD dimeric interfaces	Tm1570 dimeric interfaces
F10, 12-13 (EM), 16-17 (EI), 20-23 (YGVL), 52-58(DYQYGGG), 62-66 (VMKPE), 69Y, 88-96 (PRGEQFTQ), 99A, R113, 115-124 (EGIDDRVREL), 131-137 (SIGDFVI), 145-146 (VT), 149-150(DA), R153, 175-183 (LEYPHFTRP), 185-186 (EF), 189-191 (KKV), 195-196(LI), H200, I203, R207, T215, 218-219(NR), M222	K12, T22,M24, 27-28 (HD), 31-32 (RS), 34-37 (RTFG), 75-76 (EA), 150-163 (KPIHGVGDFNHLS), 165-166(RS), 169-170 (AI), 173-174 (DR) 176-180 (NRSFQ)

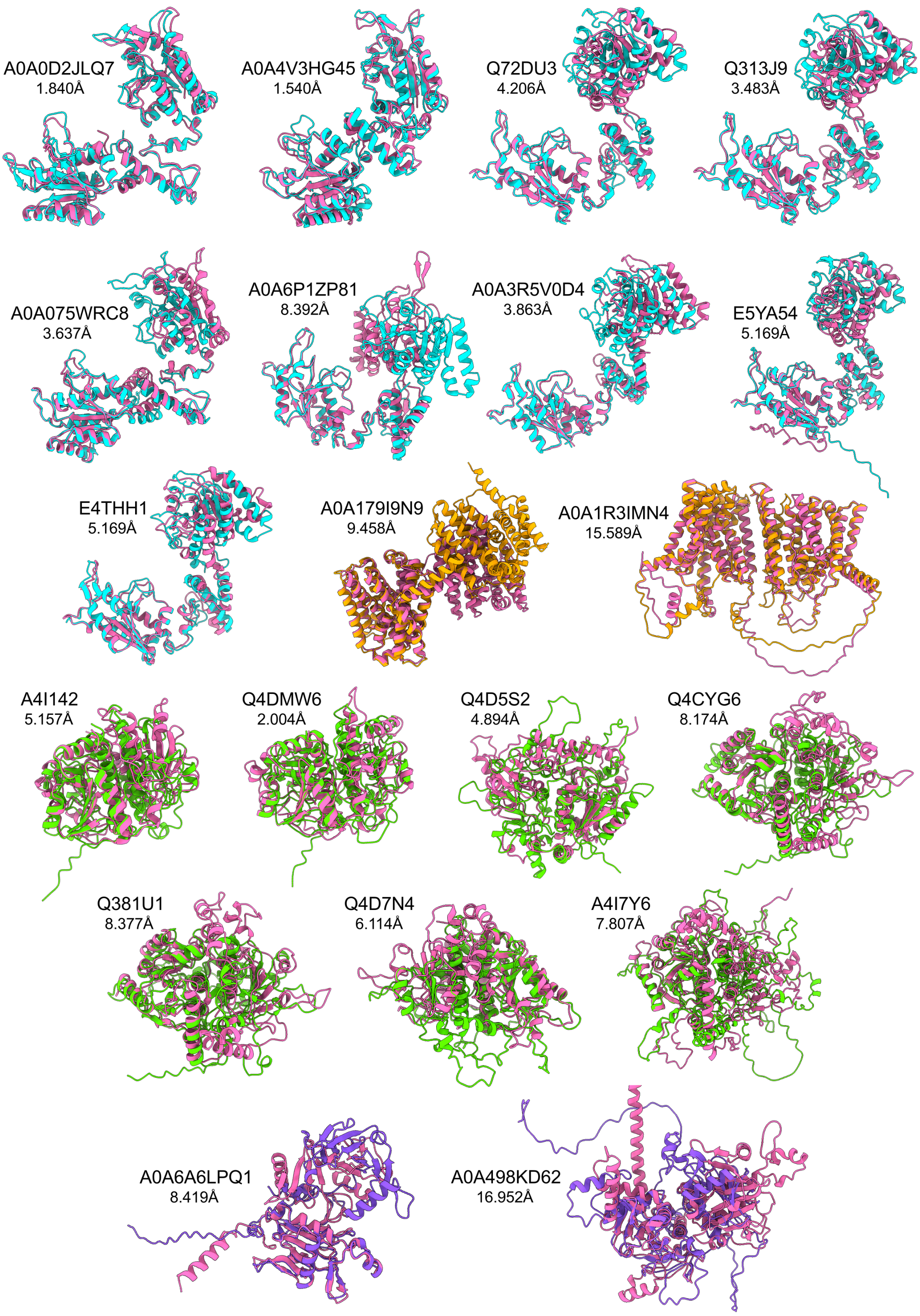

Figure S2: Superposition of models of double knotted proteins predicted by AlphaFold and RoseTTaFold. The structures from AlphaFold are colored according to the architecture: PF01746-PF09936 (cyan), PF01699-PF01699-PF01699-PF01699 (orange), PF00588-PF00588 (green), PF03587-PF03587 (purple). All RoseTTaFold predictions are colored in magenta. The Cα RMSD between corresponding structures is shown next to each pair.

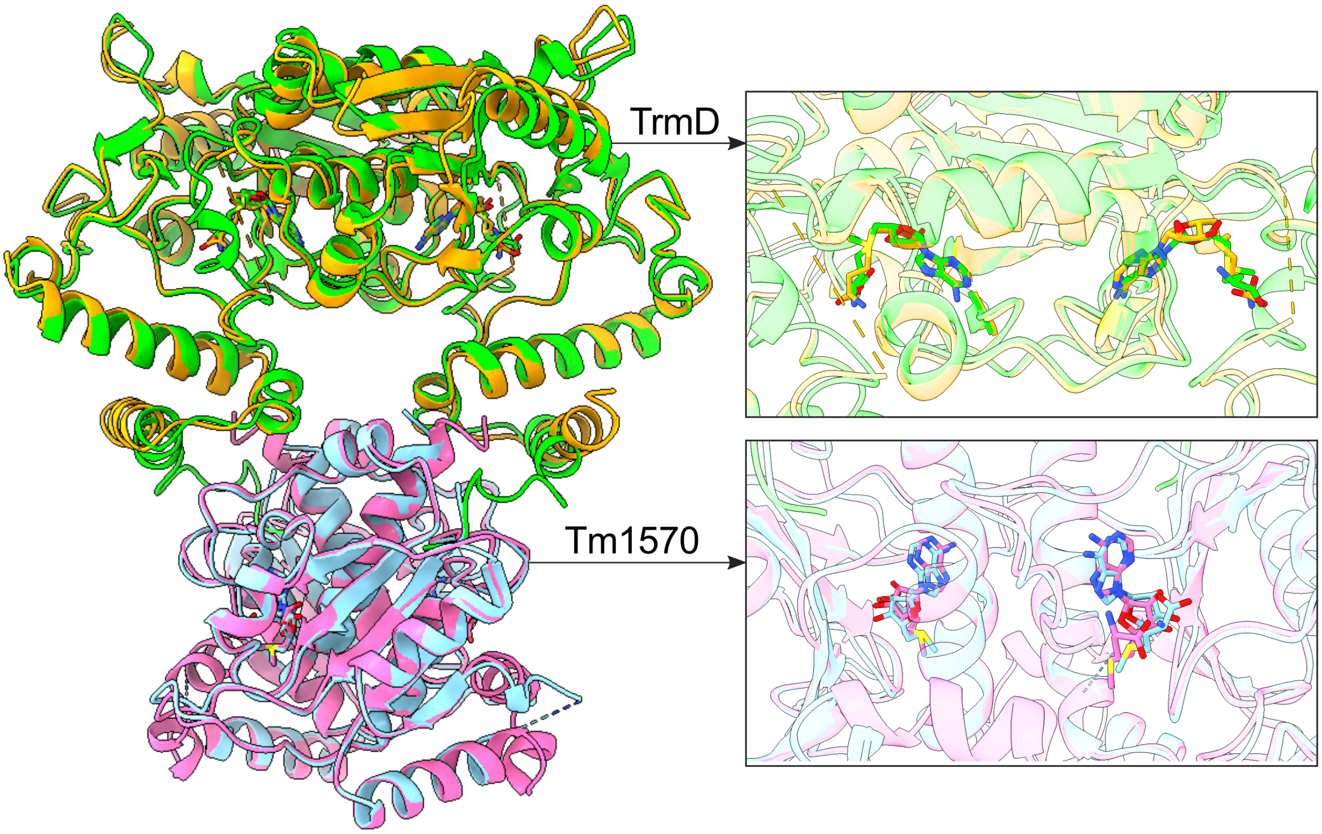

Figure S3: Superposition of SAM binding sites between the crystal structure and the fusion protein. The crystal TrmD dimer (PDB ID:5WYQ) is in yellow and the crystal Tm1570 dimer (PDB ID:3DCM) is in magenta.

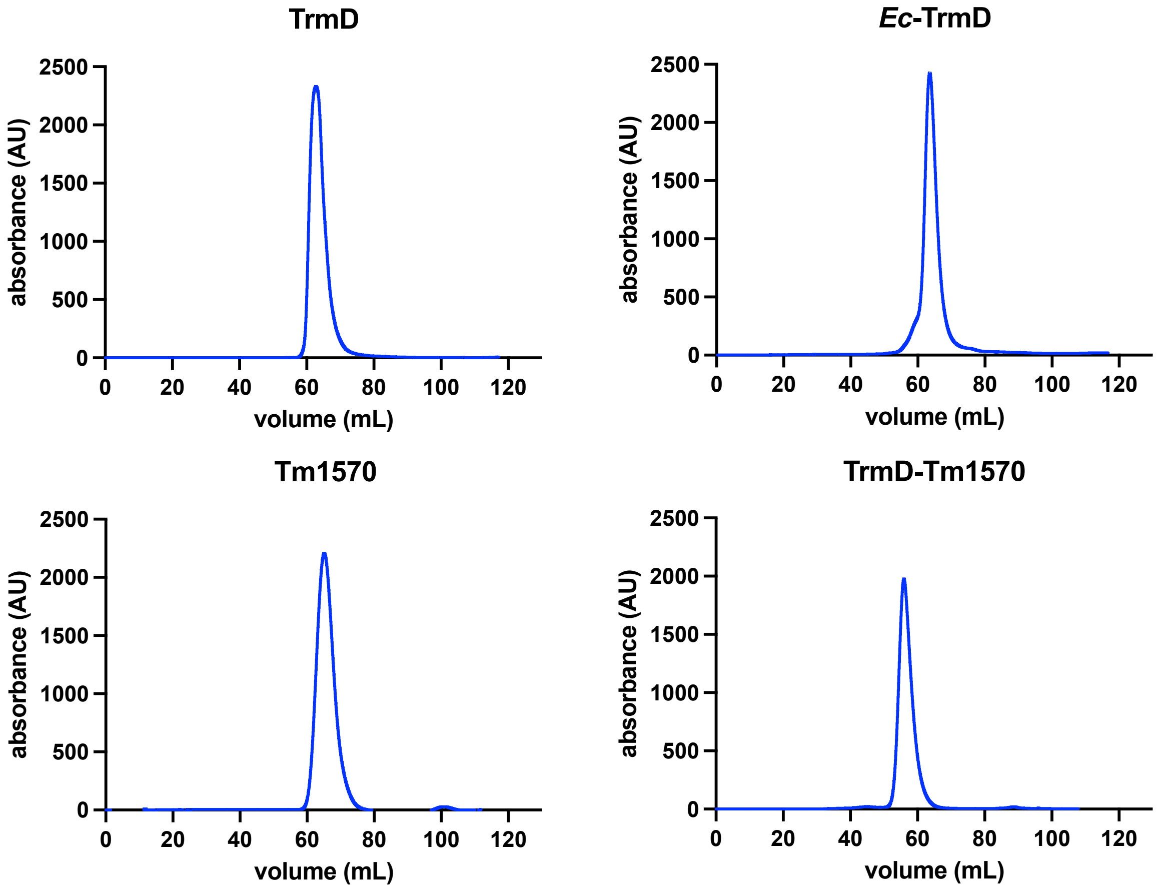

Figure S4: Size-exclusion chromatography profiles obtained for all protein constructs used in this study on the preparative grade column Superdex 75 1660 (GE Healthcare).

DNA primers for *E. coli* tRNA^{Leu} (CAG)	F1_Ec GCGACGTAATACGACTCACTATAGGCGAAGGTGGCGGAATTGGTAGA<br>R2_Ec ACTAACACCTGAAGCTAGCGCGTCTACCAATTCCGCCACCTTCG<br>F3_Ec GCGCTAGCTTCAGGTGTTAGTGTCCTTACGGACGTGGGGGTTCAAGTCCCCCCCCTCGCA<br>R4_Ec GATCCTGAATCCTGCGTGTCTACCAATTCCACCATTTGGGCCCT
DNA primers for *C. nitroreducens* tRNA^{Leu} (CAG)	F1_Cn GCGACGTAATACGACTCACTATAGGGCCCAAATGGTGGAATTGG<br>R2 _Cn GATCCTGAATCCTGCGTGTCTACCAATTCCACCATTTGGGCCCT<br>F3_Cn TAGACACGCAGGATTCAGGATCCTGTGGGGTAACACCTGTGGGG<br>R4_Cn TGCCCAAAGGGGGACTCGAACCCCCACAGGTGTTACCCCAC
DNA primers for *C. nitroreducens* 36T-tRNA	F1_Cn GCGACGTAATACGACTCACTATAGGGCCCAAATGGTGGAATTGG<br>R2_36T GATCATGAATCCTGCGTGTCTACCAATTCCACCATTTGGGCCCT<br>F3_36T TAGACACGCAGGATTCATGATCCTGTGGGGTAACACCTGTGGGG<br>R4_Cn TGCCCAAAGGGGGACTCGAACCCCCACAGGTGTTACCCCAC
Ec-tRNA	GGCGAAGGTGGCGGAATTGGTAGACGCGCTAGCTTCAGGTGTTAGTGTCCTTACGGACGTGGGGGTT CAAGTCCCCCCCCTCGCACCA
Cn-tRNA	GGGCCCAAAUGGUGGAAUUGGUAGACACGCAGGAUUCA***G***GAUCCUGUGGGGUAACACCUGUGGGG GUUCGAGUCCCCCUUUGGGCA
36T-tRNA	GGGCCCAAAUGGUGGAAUUGGUAGACACGCAGGAUUCA***U***GAUCCUGUGGGGUAACACCUGUGGGG GUUCGAGUCCCCCUUUGGGCA

Figure S5: Nucleotide sequences of tRNA substrates and DNA oligonucleotides used to produce them during the course of the *in vitro* transcription.

Table S6. The residue conservation at dimer interfaces

Residues in fusion (Calditerrivibrio nitroreducens)	% conservation
TrmD dimer	
F10	93
G56	99
P88	96
G90	100
R113	90
E115	100
G116	100
D118	100
R120	100
S131	95
G133	100
D134	89
V136	88
R153	100
P178	97
T181	90
P183	100
H200	95
I203	84
R207	92
T215	99
Tm1570 dimer	
H276	87
D277	98
R280	97
F285	84
P401	97
N410	96
H411	97
L412	99
R414	97
D422	99
R423	95

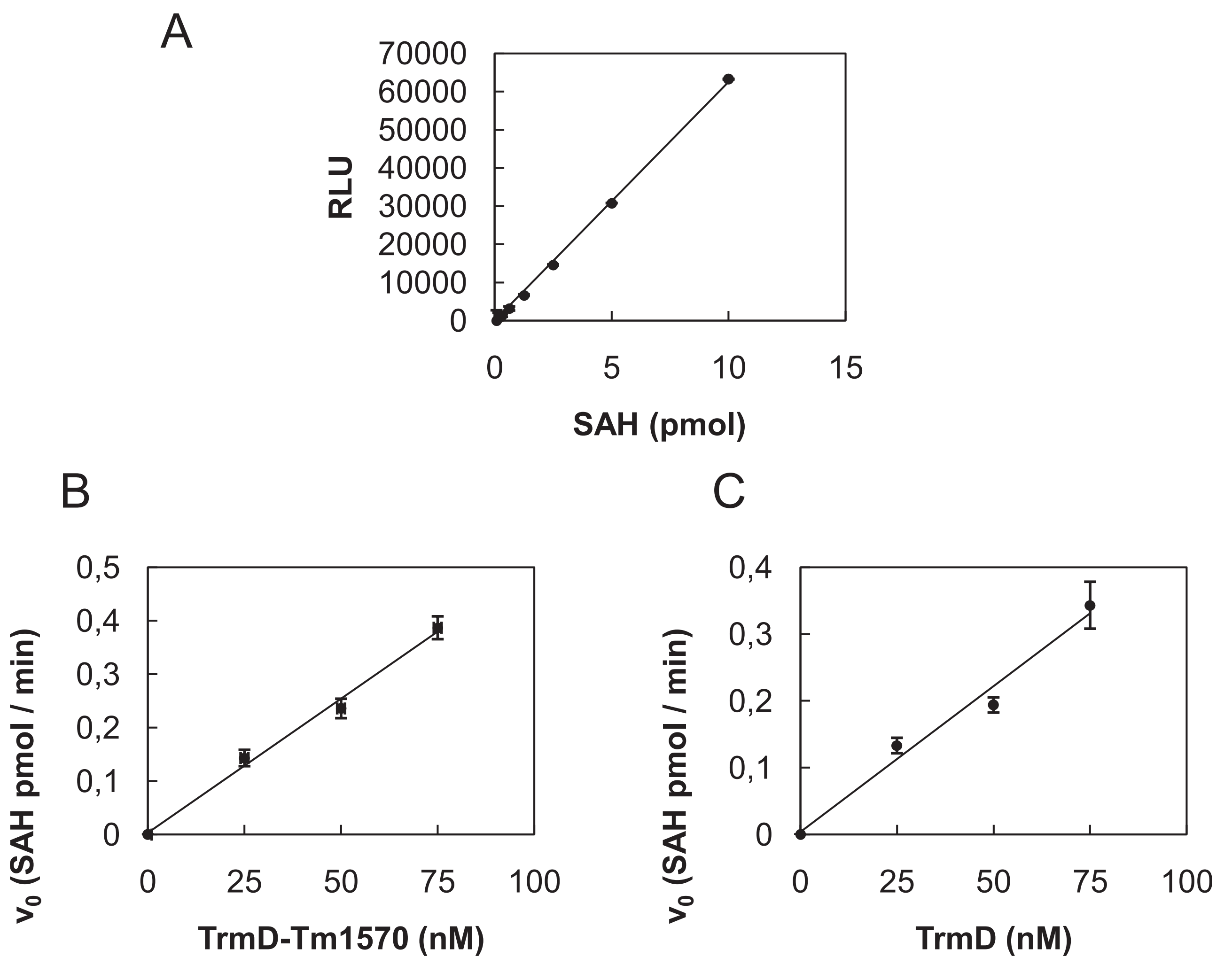

Figure S6: (A) Standard curve allowing for conversion from relative luminescence units to the amount of SAH. The linear range of the MTase-Glo detection with respect to initial velocity and concentration of TrmD-Tm1570 (B) and TrmD (C). The data points represent mean ± SD (n=3).

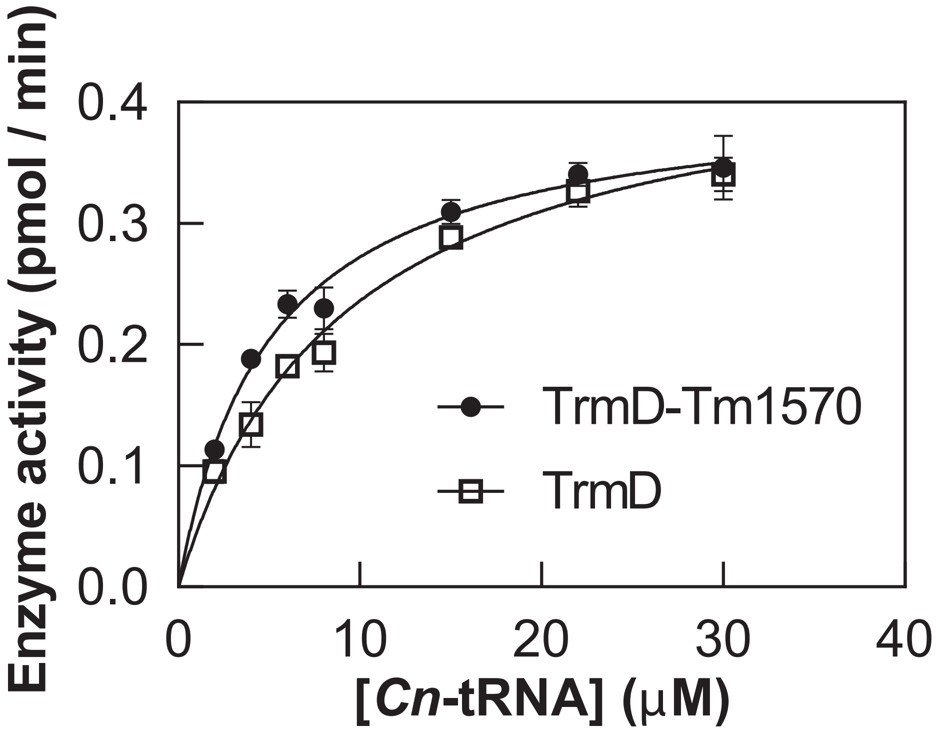

Figure S7: Michaelis-Menten plots obtained for 50 nM TrmD-Tm1570 (black circles) and 50 nM TrmD (open squares). SAM concentration was 30 μM, while tRNA concentrations in the reaction were varied from 2 to 30 μM. Values represent mean ± SD for n=3.
